# The nutrient profile and cost of specialty dietary patterns: a hypothetical case study

**DOI:** 10.1017/S1368980023002537

**Published:** 2023-12

**Authors:** Kayla-Anne Lenferna De La Motte, Caryn Zinn

**Affiliations:** Human Potential Centre, School of Sport & Recreation, Faculty of Health & Environmental Sciences, Private Bag 92006, Auckland, 1142, New Zealand

**Keywords:** Vegan, Ketogenic diet, Ultra-processed, Cost, Nutrients

## Abstract

**Objective:**

Ketogenic and vegan diets have become increasingly popular. The rising popularity of these dietary trends has been met in kind by the food industry producing a variety of specialty ultra-processed foods (UPF). Despite increasing popularity, the cost and nutrient profile of vegan and ketogenic diets (KD) that rely on UPF specialty products is poorly understood. We aimed to assess the cost and nutrient profile of vegan and KD that relied primarily on UPF and compare this to those that relied primarily on whole foods.

**Design:**

We designed and calculated the cost of four 1-d meal plans for a hypothetical weight-stable adult female. Two meal plans were created for the vegan-style and ketogenic-style diets, respectively, with one of each being predominantly whole food based and the other constituting primarily of UPF. Carbohydrates were limited to ≤50 g, protein was set at 15–20 % and fat ≥75 % for the ketogenic meal plans. Carbohydrates were set between 45 and 65 %, protein 15 and 25 % and fat 20 and 35 % for the vegan meal plans. FoodWorks dietary analysis software was used to assess data against the national Australian/New Zealand nutrient reference value for adult females and cost was calculated using Countdown online shopping (a local New Zealand supermarket).

**Setting:**

New Zealand.

**Participants:**

None.

**Results:**

The whole food-based meal plans met a greater proportion of the macro and micronutrient thresholds and were less costly when compared with the specialty-based meal plans.

**Conclusions:**

This study demonstrates that well-planned, predominantly whole food diets (regardless of dietary trend) are nutritionally superior and more cost-effective than those that rely on UPF.

The dietary approaches of veganism (VGN) and the ketogenic diet (KD) have become increasingly popular in recent years and were among the top five searched diets on Google between 2009 and 2014^(1)^. Due to VGN and KD eliminating whole food groups, it is plausible that they may be devoid of key nutrients without careful planning. Given their rising popularity, the food industry has responded with a variety of specialty products, many of which are considered ultra-processed, marketed specifically at those adhering to KD and VGN. The impact on nutrient intake, when relying primarily on ultra-processed foods (UPF), for long-term adopters of these diets is unknown and warrants further investigation.

The recent publication of the EAT-Lancet diet has propelled the consumption of ‘plant-based’ into mainstream media^(2)^. Concerns have been raised about this diet’s cost and relative affordability, particularly in middle- and low-income countries^(3,4)^. While a universal definition for VGN has not been established, it is well known as a diet that excludes all animal products and by-products including meat, fish, dairy and honey in favour of eating predominantly fruits, vegetables, wholegrains, cereals, legumes, nuts and seeds^(5)^. Studies suggest several health benefits are associated with vegan diets including a lower incidence of non-communicable diseases such as colon cancer, type 2 diabetes mellitus, obesity and non-alcoholic fatty liver disease^(6)^. There is evidence indicating that, in the absence of supplementation and fortified foods, vegan diets are devoid of key nutrients including Ca, vitamin D, vitamin B_12_ and Fe^(5,6)^. The combined insufficient intake of Ca and vitamin D could result in an increased risk for poor long-term bone health. Some evidence exists (albeit limited) to suggest that vegan diets do not contain sufficient essential fatty acids and specific recommended daily intakes may be required^(7)^. Concerns related to the application of the vegan diet in specific populations, including pregnant women and growing children, have been noted. A recent prospective observational study of vegan women indicated that they experienced a greater risk for small-for-gestational-age newborns and lower birth weight compared with children of omnivorous mothers^(8)^. Moreover, even when consuming a well-planned nutritionist-designed diet, low levels of serum vitamin A, DHA and vitamin D have been reported in a cohort of Finnish children, which could be of concern for long-term visual health^(9)^. These concerns are exacerbated by the explosion of processed meat and dairy analogues, which are often composed predominantly of soya and refined carbohydrates, and the growing availability of vegan convenience food, suggesting that such diets are not synonymous with health^(10–14)^.

The KD limits carbohydrate intake (≤50 g/d) while increasing fat intake (≥75 % total energy (TE)) with protein intake comparable to mainstream dietary patterns (15–20 % TE)^(15)^. The purpose of the KD is to establish a state of nutritional ketosis where circulating ketone bodies, predominantly *β*-hydroxybutyrate, are the main source of fuel rather than glucose^(15)^. Restricting carbohydrates below 50 g/d allows for the production of between 100 and 150 g of ketone bodies daily^(15)^. Although the KD is rooted in therapeutic clinical practice and has been used safely and effectively for the past 100 years in the treatment of intractable epilepsy and diabetes, it is becoming a popular dietary approach adopted by many to lose weight and improve health^(16–18)^. Aside from its long withstanding clinical application, the KD has been associated with favourable metabolic health outcomes in both the short and long term and recently recognised as a safe and effective treatment strategy for type 2 diabetes mellitus^(15)^. These favourable outcomes are believed to be a result of the reduction of glycation, oxidative stress and inflammation. Further research is required to ascertain the applicability of KD for the treatment of several cancers, neurodegenerative and neuro-depressive diseases but the research to date is promising^(18)^. There is limited evidence regarding the long-term adherence to a KD, an area that requires further investigation to understand the benefits and limitations of this approach for otherwise healthy individuals. Given its rising prevalence among the general population, close attention should be paid to the long-term effects on individuals living freely, especially within the context of the modern food environment where individuals have access to a wide variety of hyperpalatable UPF formulations marketed as being ‘low carbohydrate’ in an effort to ride the metaphorical health wave associated with lower carbohydrate diets. Understanding the effects of KD UPF on long-term health should be a public health priority.

There are concerns among academics and clinicians that a KD contains excessive saturated fat, insufficient fibre and is devoid of key micronutrients^(15)^. While the debate regarding the 10 % TE threshold for saturated fat is ongoing, recent advances suggest that increased dietary fat intake may not be as detrimental as previously thought^(19)^. The contention is underpinned by the saturated fat paradox of tribes, including the Maasai, Inuit and Native Americans who subsist almost exclusively on saturated fat for periods with no adverse cardiovascular outcomes^(15)^. Meanwhile, the supposition that carbohydrate-restricted diets are devoid of fibre and key micronutrients remains contentious with mixed outcomes reported among research to date^(20–22)^. Further studies are required to confirm whether long-term KD adherence negatively affects micronutrient intake and subsequent serum levels in healthy individuals. It is evident that there are potential shortcomings associated with both diets but when applied appropriately can result in improvements to health and prevention of chronic disease.

Significant resources are attributed to discovering ‘the best’ dietary pattern for longevity and health; however, it appears that studies illustrating positive outcomes have one key metric in common, a reduction in nutrient-poor UPF and a subsequent increase in whole and minimally processed foods. To date, research suggests that moving towards such healthier eating patterns (regardless of the chosen trend) is associated with a higher cost than current eating habits^(23–26)^. This is believed to largely be attributed to a reduction in the consumption of processed and UPF, which are mass produced food formulations that are cheaper than real, whole foods^(27)^. While the cost of whole foods fluctuates seasonally, processed and UPF costs remain relatively stable across the year. To date, studies predominantly investigate the cost and impact of mainstream UPF, which are known to negatively impact health^(28,29)^. Despite the increasing availability of specialty products for restrictive dietary patterns, little is known about the impact their inclusion has on diet cost, nutrient status and long-term health^(13,14,30)^. With an increasing awareness that the level of food processing affects health and limited understanding of the impact of processed specialty foods on health, it can be argued that this once niche area of nutrition requires further examination^(31)^. The purpose of this case study was to assess the nutrient profile and cost of VGN and KD under two conditions, exclusion of all UPF specialty products and inclusion of UPF predominantly specialty products, to understand the effect these products have on the nutrient status and cost of these dietary patterns.

## Methods

In this descriptive case study, four single-day hypothetical meal plans were formulated and costed for a young adult female not currently pregnant or lactating. A young adult female was chosen for this case study as women are commonly reported to be more health-conscious, therefore potentially more likely to engage in exclusionary-type diets like VGN and KD^(32)^. Demographic (height and weight) data were based on nutrient reference values threshold profiles available on the Australian National Health and Medical Research Council and New Zealand Ministry of Health (NHMRC and MOH) website^(33)^. These profiles illustrate the estimated energy requirements for individuals within given age, height and weight-bound categories. For the purposes of this case study the 19–30-year-old age category was selected alongside a height of 1·7 m and weight of 63·6 kg, this is a common time for women to begin experimenting with diets and the chosen weight and height resulted in a healthy BMI. TE expenditure was calculated using the Schofield equation, inputting the demographic data and an activity level of 1·6 (lightly active)^(34)^. TE for the four hypothetical meal plans was intended to be isocaloric to maintain current weight and set in accordance with the calculated energy needs of the hypothetical subject (±10 %). Table 1 presents the demographic data used for this case study. The vegan and KD were chosen for examination due to their rising popularity^(1)^.

**Table 1:** Case study demographics

Gender	Age (years)	Reference height (cm)	Reference weight (kg)	BMI	PAL	EER (kcal)	
Female	19–30	1·7	63·6	22	1·6	2286·6	10

PAL, physical activity level; EER, estimated energy requirement.

Two meal plans were created for VGN and KD, respectively. The following meal plans were created: whole food vegan (WFV), whole food ketogenic (WFKD), specialty vegan (SV) and specialty ketogenic (SKD). The formulation was based on popular vegan and KD meals with all recipes included in the sample diets available freely online. The vegan meal plans excluded all animal products and by-products and were formulated in accordance with the acceptable macronutrient distribution ranges (AMDR) for carbohydrates (45–65 %), protein (15–25 %) and fat (20–35 %), as per the Australian NHMRC and New Zealand MOH dietary guidelines^(33)^. While geographically specific, these guidelines closely mirror those of the UK and USA. Comparatively, the ketogenic meal plans were formulated in accordance with ketogenic guidelines for macronutrient distribution: ≤50 g carbohydrate, 15–20 % protein and ≥75 % fat^(15,21)^. When creating the whole food-based diets, predominantly foods that are unprocessed or minimally processed were selected for inclusion, while UPF were excluded where practicable. For the vegan diet, a variety of fruits, vegetables, wholegrains, nuts, seeds and legumes were included, while for the KD low-carbohydrate, high-fat foods were included with a careful selection of foods that were anti-inflammatory and reduced oxidative stress^(22)^. Examples of these foods include salmon, broccoli, berries, leafy greens, olive oil and coconut oil. When creating the specialty-based UPF meal plans, whole foods were minimised with the majority of foods selected being processed or UPF speciality marketed towards vegans and KD adopters. In both instances, foods that were convenient, acceptable and palatable were included with fresh, frozen and canned alternatives incorporated, while less commonly consumed options (like mussels and organ meats), dietary supplements such as MCT, nutritional yeast, protein powders and dietary supplement capsules or tablets were intentionally excluded. While ‘speciality products’ were those identified as being marketed specifically to a certain diet group (e.g. the packaging stated ‘vegan’ or ‘keto’), ‘ultra-processed products’ were defined in accordance with the NOVA food categorisation system. UPF are industrially processed foods that contain multiple ingredients, including additives and flavourings to create a highly palatable product with a long shelf life. Typical mainstream examples of UPF include sugary drinks, packaged snacks, frozen dinners and fast foods. Nutrition information for the specialty products was gathered from Countdown (a local New Zealand supermarket) online shopping website. Meal plans were analysed using FoodWorks Professional V.10 (Xyris software), which uses an Australian and New Zealand food database, a commonly used nutrient analysis software^(20,22,35)^.

The cost for each meal plan was calculated by the price per weight of food consumed^(35)^. Items on special and home brands were not included; instead, popular brands were chosen. Free-range and grass-fed products were selected over traditionally farmed alternatives where possible. Cost data were gathered using Countdown (a local New Zealand supermarket) online shopping website.

## Results

The vegan and KD meal plans and corresponding nutrient analyses are presented in Tables 2 and 3, respectively. All four meal plans met energy requirements. The WFV, WFKD and SKD meal plans met respective dietary macronutrient guidelines while the SV diet contained insufficient carbohydrate in accordance with the AMDR (41·7 %) and insufficient protein in accordance with the AMDR (11·6 %) but sufficient protein when using the g/kg/body weight recommended daily intake threshold (1 g/kg/body weight).

**Table 2:** Vegan and ketogenic sample meal plans

WFV	SV	WFKD	SKD
Breakfast Oats: ¼ cup rolled oats, 1 medium (120 g) green apple, 2 Tb hemp protein, 1 Tb LSA, ¼ cup almond milk, ½ tsp nutmeg, ½ tsp cinnamon, 1 Tb chia seeds, 2 Tb frozen blueberries, 1 brazil nut, coffee made with ½ cup soya milk	Breakfast Vegan granola bowl: 80 g Blue Frog vegan-certified granola, 2 Tb vegan honey, 1 cup oat milk, 20 g abundance plant cake, 1 medium (80 g) banana, coffee made with ¼ cup soya milk	Breakfast Vegetable omelette: 100 g (2 large) eggs, 95 g avocado, 1 Tb butter, 90 g baby spinach, coffee made with 15 ml cream and 20 ml regular full fat milk	Breakfast Keto granola bowl: 85 g Mighty Food Kitchen granola, 5 g monk fruit sweetener, 130 g Greek yogurt, 34 g frozen raspberries, 23 g Ceres organics coconut smiles
Lunch Vegan Buddha bowl: ½ cup brown rice, ½ cup broccoli, ¼ cup chickpeas, 2 Tb mashed avocado, ¼ cup red cabbage, ¼ cup baby spinach, ¼ cup steamed butternut, 1 tsp tahini, ¼ tsp paprika, ¼ tsp turmeric, 1 Tb olive oil, ¼ cup red capsicum	Lunch Heat and eat curry: Just heat and eat vegan chickpea curry with rice (400 g)	Lunch Chicken salad: 25 g cos lettuce, 58 g rocket, 90 g spiralled raw zucchini, 35 g walnuts, 130 g chicken breast, ½ tsp olive oil, 1 tsp salt, 57g Greek yogurt, ½ Tb lemon juice and 2 garlic cloves	Lunch Bacon and egg sandwich: 57 g (2 slices) Home St keto bread, 80 g bacon rashers, 80 g (2 large) fried eggs, 20 g mayonnaise, 36 g mixed nuts, 520 ml commercial kombucha
Dinner Lentil and tempeh curry: ¼ cup brown lentils, ½ cup red lentils, 2 cups silverbeet, ½ cup carrots, 1 cup canned tomato, ¼ cup coconut cream, ½ cup tempeh, ½ cup potato, ¼ cup diced onion, ¼ tsp cumin, ¼ tsp curry powder, ¼ tsp garam masala, ½ tsp iodised table salt, 1 tsp Cocavo oil	Dinner Beyond burger and fries: 1 beyond meat free burger patty, 1 white hamburger roll, 2 Tb vegan mayonnaise, 50 g vegan cheddar cheese, 30 g sliced tomato, ½ cup shredded lettuce, 100 g frozen French fries	Dinner Cabbage lasagne: 90 g white cabbage, 20 g diced white onion, 1 garlic clove, 1 Tb butter, 1 Tb cream cheese, ¼ cup shredded cheddar cheese, 200 g beef mince	Dinner Keto spaghetti and meatballs: 100 g Slendier konjac noodles, 200 g Hellers meatballs in sauce
Snacks Dried fruit and nut butter: ½ cup dried figs, 1 Tb cashew nut butter	Snacks Snack bars: 1 Tasti lamington snack bar, 1 abundance plant cake	Snacks Coconut yogurt bowl: 60 g coconut yogurt, 1·5 Tb sunflower seeds, 1 Tb pumpkin seeds, 4 Tb chia seeds, ¼ cup frozen blueberries	Snacks Keto crackers and cheese: 40 g Penati nut and seed crackers, 30 g shredded cheddar cheese

T, tablespoon; tsp, teaspoon.

**Table 3:** Nutrient analysis of vegan and ketogenic meal plans

Nutrient	NRV/vegan goal	WFV	SV	NRV/keto goal	WFKD	SKD
Energy (calories)	2286·6	2421·2	2285·4	2286·6	2541·8	2523·7
Carbohydrate (g)	257·2–371	278·5	279·6	≤50‡	22·9	49·7
%TE	45–65‡	45·2	41·70	≤10	3·5	7·7
Protein (g)	85·7–143	95·5	65·3	85·7–143	142·8	125
%TE	15–25‡	16	11·6	15–25	22·8	20·1
g/kg/body weight	0·75	1·5	1	0·75	2·2	1·9
Fat (g)	50·8–88·9	89·7	81·7	139·7–190·5	201·3	199·9
%TE	20–35‡	32·8	31·6	55–75‡	70	70
Saturated fat (g)	25·4	21·8	43·2	25·4	80·2	69·6
%TE	10	8·1	17	10	28·4	24·4
Trans fat (g)	<2·4	0·1	0·2	<2·4	4·1	2
%TE	<1*	0·1	0·1	<1*	1·4	0·7
Linoleic acid (O6 PUFA) (g)	8†	19·3	4·2	8†	28·3	13
*α*-linolenic acid (O3 PUFA) (g)	0·8†	5	0·2	0·8†	13	1·2
*n*-6: *n*-3 ratio	10	3·9	21	10	2·2	10·8
Fibre (g)	25†	80	27·9	25†	41·1	78·2
Total sugar (g)	50	101·5	52·2	50	17·1	29·8
%TE	≤10	16·8	9·1	≤10	2·7	4·7
Thiamine (mg)	1·1	2	0·4	1·1	1·1	0·9
Riboflavin (mg)	1·1	1·7	0·4	1·1	1·8	1·6
Niacin (mg)	14	20·6	3·5	14	32·4	5·5
Vitamin C (mg)	45	207·2	20	45	146·1	9·9
Vitamin A (μg)	700	2464·6	73·4	700	1766·7	245·1
Vitamin E (mg)	7†	33·9	3·4	7†	16·5	17·9
Vitamin B_12_ (μg)	2·4	1·8	0·6	2·4	6·8	2·8
Folate, total (μg)	400	680·9	134·8	400	645	137·6
Ca (mg)	1000	1052	531·5	1000	1141·8	794·8
Fe (mg)	18	26·9	2·9	18	18·2	8·7
Mg (mg)	310	998·4	137·6	310	637·2	166·7
Zn (mg)	8	16·1	1·7	8	21·4	11·3
Na (mg)	460†	1926	2453·2	460†	2697·7	3604·7
K (mg)	2800†	5156	783·8	2800†	3805·6	2037·8
P (mg)	1000	1877	252·4	1000	2185·6	1812·4
Se (μg)	60	89·3	12·2	60	132·9	76·4
I (μg)	150	264·8	77·3	150	895·7	128·4

NRV, nutrient reference value; RDI, recommended daily intake.

* WHO recommendation for trans fat.

† AI were used as RDI were unavailable.

‡ Mainstream AMDR used as guidelines for vegan sample diets and ketogenic specific guidelines used for ketogenic sample diets.

The WFKD met all micronutrient thresholds while the SKD did not meet several micronutrient thresholds (thiamine, niacin, vitamin C, vitamin A, folate, Ca, Fe and Mg). The WFV diet met all but one micronutrient threshold (vitamin B_12_), while the SV diet failed to meet any of the micronutrient thresholds. Fibre content was sufficient, meeting adequate intakes across all sample meal plans. The WFV meal plan contained more sugar than the SV meal plan (16·9 % and 9·1 %, respectively) while the WFKD meal plan contained less sugar than the SKD meal plan (2·7 % and 4·7 %). The WFV meal plan contained less saturated fat than the SV meal plan (8·1 % and 17 %, respectively) while the WFKD meal plan contained more saturated fat than the SKD meal plan (28·4 % and 24·4 %, respectively). Na intake surpassed the suggested dietary intake in three of the four meal plans (SV, WFKD and SKD).

**Fig. 1:**
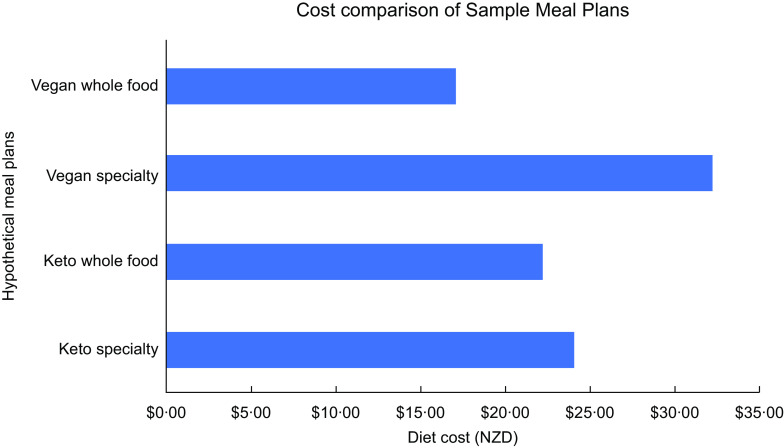
Cost analysis of vegan and ketogenic meals plans

Cost comparison for the four 1-d meal plans is presented in Fig. 1. Cost differential was most pronounced between the WFV ($17·04) and SV diets ($32·19). The WFKD ($22·24) and SKD ($24·07) diet costs were similar.

## Discussion

The results of this case study illustrate that, regardless of dietary approach, micronutrient status and cost are negatively impacted by the inclusion and reliance on UPF specialty products. The whole food-based meal plans had a more favourable nutrient profile and cost less for both vegan and KD. The vegan meal plans contained the highest quantity of sugar (likely the result of greater carbohydrate content), beyond the WHO 5 % TE recommendation. In accordance with previous findings, a well-formulated whole food-based vegan diet met all requirements, except vitamin B_12_^(6)^. This is expected given the absence of animal foods, which are rich in vitamin B_12_. Comparatively, the SV meal plan contained the lowest total calories, insufficient protein (in accordance with AMDR) and inadequate levels of all key micronutrients. Concerns related to protein content and micronutrient status of vegan diets have previously been raised. When nutrient-dense foods such as meat and animal by-products are excluded from the diet, a larger total volume of food may need to be consumed to meet nutrient requirements, which may pose a challenge due to the satiating properties of high-fibre plant foods^(36)^. Moreover, when there are fewer calories available, it becomes challenging to meet requirements based on larger total calorie intakes. The WFKD meal plan met all requirements, a finding similar to that of a randomised control trial of obese adults on a KD and a recent case study analysis of low-carbohydrate diets^(21,22)^. Three of the four meal plans in the present case study exceeded the suggested daily threshold for Na (2000 mg).

There are differences between the two dietary patterns that warrant further discussion. Given the contention surrounding fibre, protein, saturated fat, micronutrients and their bioavailability and cost, these topics will be explored in further detail.

### Fibre

Fibre consumption, which is known to support vascular and gut health, has been associated with a lower incidence of CVD. Population-based studies commonly report low intake of fibre, usually attributed to inadequate fruit and vegetable consumption^(5)^. This case study showed that fibre requirements can be achieved with vegan and KD. The WFV meal plan contained more fibre than the SV meal plan (80 g and 27·5 g, respectively). This finding is in accordance with previous literature that illustrates vegans typically surpass fibre recommendations^(5,6,38,39)^. The higher intake of fibre in the WFV meal plan is due to the variety of plant foods included in comparison with the UPF speciality products in the SV meal plan. While fibre has been highlighted as a potential concern with the KD, both the current case study and previous findings suggest that ample fibre intake can be achieved through the inclusion of a variety of nuts, seeds and vegetables^(15,22)^. The WFKD contained 41·1 g of fibre, while the SKD contained 78·2 g. The Hellers meatballs in sauce (dinner meal) were the greatest contributor with 29·60 g of fibre in a 200 g serving. This is surprising – as one would have expected the konjac noodles to be the greater provider of fibre; however, this is likely due to a low-carbohydrate filler like psyllium husk used in this product. The net carbohydrate content of this product is ∼3 g/200 g serving with ∼10·2 g of fibre. These findings are vastly different to those of Bracci *et al.* ^(20)^ whose case study results suggest that achieving sufficient fibre intake within the confines of the KD requirements is not possible. This inconsistency illustrates the impact that the types of foods chosen are an important factor in achieving sufficient fibre intake. The same can in fact be said for all nutrients, which is why careful planning and understanding the limitations and nutrients that may be challenging to obtain in each diet are important knowledge for adopters. These findings suggest that sufficient fibre intake can be achieved with vegan and the KD, which, given the importance of fibre for gut and vascular health, is important.

### Protein

This study illustrates that whole food-based diets contain more protein than those that rely on UPF specialty products. The WFV and WFKD contained more protein (95·5 g and 142·8 g) than the SV and WKD meal plans (65·3 g and 125 g). As a result, three of the four meal plans contained sufficient protein in accordance with AMDR, with the SV meal plan not meeting the 15 % threshold. Previous research suggests that those adhering to vegan diets may be at risk of consuming insufficient protein and select essential amino acids (including tryptophan)^(22,36)^. This is likely the result of the exclusion of animal products and by-products in the SV meal plan, which readily provide complete sources of protein containing all essential amino acids^(6)^. For vegans, adequate protein intake can be achieved through careful consideration and the consumption of a variety of whole foods^(5)^. This is shown in the present case study with the WFV diet containing 95·5 g or 16 % protein. When variety is reduced in favour of energy-dense specialty convenience products, protein content becomes a concern, with the SV diet containing 65·3 g or 11·6 % protein. It is also worth noting that the bioavailability of plant proteins is variable (50–70 %) and further affected by heating and cooking^(6)^. Moreover, due to their relatively new introduction to the market, the digestibility of meat analogues is unknown and requires further study to understand^(12)^. These findings highlight the importance of a well-planned diet to achieve a specific macronutrient intake. When working with individuals adhering to a vegan diet, nutrition professionals can consider the inclusion of a plant-based protein supplement (e.g. hemp or pea protein) to enhance protein consumption should this be needed. It is worth noting that while a ‘food-first’ approach is often favoured, oftentimes when excluding key nutrients, the inclusion of supplements can enhance overall nutrient intake and make meeting thresholds more attainable.

### Saturated fat

Saturated fat intake was beyond the 10 % TE threshold as recommended by the WHO in both the WFKD (28·4 %) and SKD (24·4 %) and the SV (17 %) meal plans^(33)^. Based on previous research, and the nature of the KD, higher intakes of saturated fat were not unexpected with this dietary trend^(20,22)^. Although there is controversy and ongoing debate as to whether increased saturated fat intake negatively affects cardiovascular health, recent advances suggest that within the context of a whole food low-carbohydrate diet, saturated fat intake is not a key concern^(15,19)^. There is an acknowledgement among academics that food matrices and the overall dietary trend should be considered rather than isolating nutrients as this is not reflective of real life and the way that people consume food^(40)^. In comparison, vegan diets are usually associated with lower intakes of saturated fat and higher intakes of carbohydrates (due to a reliance on fruits, vegetables, grains, cereals and legumes)^(5,6)^. However, with an increasing reliance on meat analogues, recent studies have shown an increase in saturated fat intake when convenience-type vegan diets are followed, illustrating the need to further understand the impact of food quality on health regardless of dietary pattern^(13,14)^. Given these findings, it is important that future research aims to quantify the effect of saturated fat on health in context of a high carbohydrate, predominantly processed food diet.

### Vitamins and minerals

Both speciality meal plans (SV and SKD) did not meet Ca, vitamin A or Fe thresholds. Ca, alongside vitamin D, is crucial for maintaining bone health and preventing osteoporosis. Both Ca and vitamin D (which is affected by both nutrition and seasonal sunlight exposure) are commonly reported to be lower than recommended daily intakes in vegans^(36,38,41,42)^. Bioavailability and Ca absorption from plant-based foods is variable (5–50 %) vegans should aim for low-oxalate foods, including kale and bok choy, to maximise Ca absorption^(5)^. To prevent vitamin D and Ca deficiencies, which can increase risk of fractures and have long-term health consequences (especially in females approaching menopause), the addition of a suitable supplementation regime on advice from a health professional can be considered. It should be noted that the inclusion of high-quality nutritional supplements would likely increase overall diet cost. Vitamin A (found predominantly in red and orange vegetables like carrots and bell peppers, beef liver, fish oil, milk and eggs) is crucial for supporting vision, immune function, reproduction and growth and development. A lack of whole foods in the specialty UPF meal plans is the likely cause of this observed shortfall and illustrates that relying on UPF can negatively impact nutrient intake, regardless of overarching dietary principles followed.

Fe is commonly reported as a nutrient of concern for vegans with absorption from non-haem sources varying from 1 to 23 % depending on Fe status and the presence of dietary enhancers and inhibitors^(5,6)^. With this knowledge, health-conscious vegans regularly supplement with Fe, pay careful attention to Fe absorption enhancers and inhibitors and closely monitor Fe status. It is worth noting that not all adopters of the vegan diet are motivated by health and that those who rely on UPF are at risk of falling short of Fe requirements. This could easily be corrected by including more whole foods. Fe intake in the SKD could be easily enhanced through the inclusion of green-lipped mussels or organ meats such as liver. These food sources were intentionally excluded from the sample meal plans as they are seen to be an acquired taste, but small amounts (i.e. 5 g) would suffice and organ meats could be frozen and grated into meals like bolognaise to enhance acceptability^(22)^. Consumption of organ meats could also reduce overall diet cost due to their relative cost-effectiveness as an animal protein source.

When considering the shortfall of vitamin E, Zn and ALA in the SV plan, this is likely the result of a lack of whole food. This would be the reality for anyone relying predominantly on specialty UPF as the basis of their diet. To rectify these nutrient shortfalls for the SV and SKD plan, a supplement could be included or (in our opinion the more favourable approach) diet quality could be improved to include more whole foods. This illustrates the importance of diet quality in achieving optimal nutrient intakes.

### Ultra-processed foods and health

The seemingly ever-increasing availability of UPF (both generic and speciality) is a public health concern as the consumption of these products in large quantities negatively affects health. A recent prospective longitudinal cohort study found that with each 10 % increment increase in UPF consumption, there was a corresponding 15 % increase in the risk of all-cause mortality^(45)^. While this research is based on mainstream UPF, given the similarities between these and niche or speciality foods, it is likely that these outcomes are transferable. There is a clear need for further exploration of the metabolic effects of a predominantly UPF diet, in the context of specialty dietary trends, with consideration on how satiety signalling is affected by elevated consumption of such products. Current evidence suggests a clear association between UPF consumption and non-communicable diseases; while the mechanisms at play are not yet known, due in part to the relatively recent introduction of these food formulations, it is believed to be multifaceted. First, these foods are formulated to be palatable and often include a combination of fat, sugar and salt, which drives overconsumption and is said to be linked to food addiction^(28)^. Due to manufacturers’ desires to enhance shelf life and promote long-term stability, the fats found in these foods are hydrolysed industrial trans fats, known to be harmful to health and promote inflammation^(46)^. It can be argued that replacing sugar with non-nutritive artificial sweeteners reduces overall calories and curbs consumption, but the evidence on this appears mixed^(47)^. Moreover, the potential implications of non-nutritive artificial sweeteners for gut health remain poorly understood. Second, processing and cumulative food additives appear to promote carcinogenicity and genotoxicity, adversely affecting the expression of genes and increasing risk of cancer^(28)^. Finally, high intakes of UPF adversely affect the gut microbiome, with evidence suggesting that they increase C-reactive protein and lipoprotein levels^(28)^. The inflammatory effect of UPF has received much attention in recent years; however, exact mechanisms of action require further exploration.

It is worth noting that when considering UPF, factors beyond the food formulations themselves are worth exploring, this includes but is not limited to the impact of additives and increased exposure to harmful chemical substances, which might cause endocrinological disturbances through both processing and packaging^(48,49)^. Prolonged intake of an UPF diet is associated with biochemical alterations including oxidative stress, inflammation and intestinal dysbiosis as well as impaired immunological health^(50)^. Research illustrates that chemicals in food packaging migrate to the foods we eat when they are in direct contact with one another, these are known as food contact chemicals and many have been linked to adverse health consequences^(48)^. Toxicity thresholds are determined based on singular chemical components, this means that analyses do not take into account the cumulative impact of several different chemicals and their exposure^(49)^. Moreover, these thresholds are determined based on the chemical component but do not consider the by-products created during heating, cooling and storing for long periods of time which may also be toxic^(49)^. Finally, these toxic thresholds do not account for the effects which may exist at lower doses, particularly to the endocrine system^(48,49)^. It is evident that the negative impacts of UPF are far reaching, beyond the nutrients themselves.

### Cost

The whole food-based meal plans were cheaper than the specialty UPF meal plans for vegan and KD. The WFV and WFKD cost $17·04 and $22·24, respectively, while the SV and SKD cost $32·19 and $24·07, respectively. The cost differential is the result of the pre-prepared foods included in the specialty UPF meal plans like the frozen heat-n-eat meal for the SV ($13·00) and the pre-made meatballs in sauce for the SKD. The cost of the SV meal plan could be reduced by including cheaper convenience options like canned ‘nut meat’ and ramen noodles rather than pre-prepared options. However, it is worth considering that research suggests that consumers purchasing habits are trending towards an increased consumption of convenience foods such as meat analogues and heat-and-eat meals and away from singular items to prepare their own meals^(11,12,14)^. The KD meal plan cost was comparable for both the WFKD ($22·24) and SKD ($24·07), likely because there are not as many convenience options available for this dietary trend and both meal plans contain multiple meat and dairy products. The cost of the KD is poorly researched, to date there are no studies examining their cost and only two examining the cost of low-carbohydrate healthy fat diets^(35,44)^. The findings of this case study are in accordance with previous research, indicating that lower carbohydrate diets tend to be more costly than regular diets. With the growing popularity of this dietary trend, further work should be conducted to assess the cost of this dietary approach.

The costs of both the KD and VGN diets could have been reduced by applying simple budgeting guidelines such as including items on sale or non-branded items, by pricing items at green grocers and butchers rather than from a supermarket exclusively and by shopping seasonally. Eating a predominantly whole food diet would reduce cost in this instance by reducing or removing specialty products. Interestingly, these findings contradict research regarding the cost of mainstream (i.e. not vegan or KD) UP foods, which suggests that high-quality diets are more expensive than low-quality diets^(25)^. This may be in part due to the fact that VGN and KD specialty products are not yet produced on the same scale as mainstream UP foods; however, research indicates that for meat analogues at least this will not be the case for much longer^(12–14)^.

## Conclusion

This case study highlights the importance of diet quality, regardless of the overall dietary pattern. There are several key limitations. First, despite the methods being used in other research, these hypothetical case studies are indicative of a single day. We believe that the meal plans are reflective of the eating patterns of those adhering to VGN and KD diets as they include realistic and familiar foods and the author’s own unpublished work found the foods included here are commonly consumed by those adhering to the vegan and KD. Second, this case study reflects intakes rather than serum levels. Serum levels may be more accurately indicative of the nutritional impacts of dietary trends. While they are not commonly measured in studies to date, predominantly due to associated costs, resources and the burden on participants, they might be an important consideration for future studies. Furthermore, meal plans were only created for a single demographic. Given anyone could adopt these dietary patterns, it is worth considering whether these diets would meet the nutrient requirements of populations other than non-pregnant adult females. Particularly, it is worth considering the applicability of these dietary patterns for children, pregnant women and the elderly given these populations require specific nutritional considerations.

Despite these limitations, this work highlights the importance of considering diet quality regardless of the perceived healthiness of a given dietary trend and illustrates a need to further understand the impact that specialty products have on nutrient density, nutrient intake, long-term health and the cost of food. Given the rising popularity of these types of products, it is a key public health consideration to establish whether these products are safe for long-term consumption. While there are evident, and widely reported differences between the KD and vegan diets, the purpose of this article is not to pit ketogenic and vegan approaches against one another but rather to highlight that the level of processing a food has undergone is an important consideration given the rapid changes in the modern food environment and the expected changes in coming years. Public health agencies should educate consumers to ensure they are choosing nutrient-rich and cost-effective food options. This choice will directly affect their risk of all-cause mortality and promote a longer more healthful life. While fortification is increasingly common, especially among speciality products, consumers should not rely on fortification for adequate micronutrient intake. Instead, public health agencies should equip consumers with adequate knowledge of the best whole food sources of various vitamins and minerals, as the bioavailability of micronutrients in fortified foods is not clearly established.

We do not advocate for a one-size-fits-all approach to nutrition and recognise the importance of context and that preferences, culture, religion and socio-economic factors all play a part in an individual’s dietary choices. We do believe that food quality, regardless of the chosen dietary trend, is an important consideration and that whole, unprocessed foods should form the basis of any diet, whether it be vegan, ketogenic or any other chosen approach.
